# Cytokine clearance with CytoSorb® during cardiac surgery: a pilot randomized controlled trial

**DOI:** 10.1186/s13054-019-2399-4

**Published:** 2019-04-03

**Authors:** Elettra C Poli, Lorenzo Alberio, Anna Bauer-Doerries, Carlo Marcucci, Aurélien Roumy, Matthias Kirsch, Eleonora De Stefano, Lucas Liaudet, Antoine G Schneider

**Affiliations:** 1 Adult Intensive Care Unit, Centre Hospitalier Universitaire Vaudois, Lausanne, Switzerland; 2 Division of Haematology and Central Haematology Laboratory, Centre Hospitalier Universitaire Vaudois, Lausanne, Switzerland; 3 Department of Anaesthesiology, Centre Hospitalier Universitaire Vaudois, Lausanne, Switzerland; 4 Departement of Cardiovascular Surgery, Centre Hospitalier Universitaire Vaudois (CHUV), Centre Hospitalier Universitaire Vaudois, Lausanne, Switzerland; 5 Faculty of Biology and Medicine, University of Lausanne, Lausanne, Switzerland

**Keywords:** Haemoadsorption, Cardio-pulmonary bypass, Cytokines, Coagulation factors, CytoSorb®

## Abstract

**Background:**

Cardiopulmonary bypass (CPB) is often associated with degrees of complex inflammatory response mediated by various cytokines. This response can, in severe cases, lead to systemic hypotension and organ dysfunction. Cytokine removal might therefore improve outcomes of patients undergoing cardiac surgery. CytoSorb® (Cytosorbents, NJ, USA) is a recent device designed to remove cytokine from the blood using haemoadsorption (HA). This trial aims to evaluate the potential of CytoSorb® to decrease peri-operative cytokine levels in cardiac surgery.

**Methods:**

We have conducted a single-centre pilot randomized controlled trial in 30 patients undergoing elective cardiac surgery and deemed at risk of complications. Patients were randomly allocated to either standard of care (*n* = 15) or CytoSorb® HA (*n* = 15) during cardiopulmonary bypass (CPB). Our primary outcome was the difference between the two groups in cytokines levels (IL-1a, IL-1b, IL-2, IL-4, IL-5, IL-6, IL-10, TNF-α, IFN-γ, MCP-1) measured at anaesthesia induction, at the end of CPB, as well as 6 and 24 h post-CPB initiation. In a consecutive subgroup of patients (10 in HA group, 11 in control group), we performed cross-adsorber as well as serial measurements of coagulation factors’ activity (antithrombin, von Willebrand factor, factor II, V, VIII, IX, XI, and XII).

**Results:**

Both groups were similar in terms of baseline and peri-operative characteristics. CytoSorb® HA during CPB was not associated with an increased incidence of adverse event. The procedure did not result in significant coagulation factors’ adsorption but only some signs of coagulation activation. However, the intervention was associated neither with a decrease in pro- or anti-inflammatory cytokine levels nor with any improvement in relevant clinical outcomes.

**Conclusions:**

CytoSorb® HA during CPB was not associated with a decrease in pro- or anti-inflammatory cytokines nor with an improvement in relevant clinical outcomes. The procedure was feasible and safe. Further studies should evaluate the efficacy of CytoSorb® HA in other clinical contexts.

**Trial registration:**

ClinicalTrials.gov NCT02775123. Registered 17 May 2016.

**Electronic supplementary material:**

The online version of this article (10.1186/s13054-019-2399-4) contains supplementary material, which is available to authorized users.

## Background

Cardio-pulmonary bypass (CPB) is routinely used throughout the world during open-heart surgery. This procedure is associated with a complex inflammatory response through the activation of both coagulation and alternative pathway of the complement system. Numerous inflammatory molecules (C3a, C5a, histamine, IL-6, IL-8, TNFα) are released and activate cellular response leading to systemic inflammation, increased vascular permeability and thrombosis [1–4]. When severe, this phenomenon might lead to systemic hypotension and organ dysfunction, a situation referred to as “post-pump syndrome” [4]. Given the association between elevated pro-inflammatory cytokine levels and adverse clinical outcomes (post-operative acute kidney injury (AKI) [5], decreased systemic vascular resistance [6] and reduced lung function [7]), it has been postulated that extra-corporeal removal of cytokines could improve outcomes.

CytoSorb® (CytoSorbents Corporation, Monmouth Junction, NJ, USA) is a recent haemoadsorption (HA) device designed to remove cytokines from the blood. CytoSorb® cartridges contain biocompatible sorbent polystyrene divinylbenzene beads coated with polyvinylpyrrolidone, capable of removing middle molecular weight molecules using a combination of hydrophobic interactions and size exclusion [8–11]. These cartridges can easily be inserted in a cardio-pulmonary bypass circuit.

To date, little is known about CytoSorb®’s efficacy during cardiac surgery [9]. Small case-control series have suggested improved clinical outcomes associated with CytoSorb® HA during heart transplantation [12], surgical management of acute infective endocarditis [13] or in patients with severe post-CPB systemic inflammation response syndrome [14]. A single pilot randomized controlled trial has compared intra-operative CytoSorb® HA with standard CPB in 32 patients undergoing elective cardiac surgery [15]. In this trial, in patients with low to medium risk of complications, the procedure was associated neither with a decrease in peri-operative cytokine levels nor with an improvement in clinical outcomes.

We have therefore designed a pilot randomized controlled trial to evaluate the potential of CytoSorb® HA to decrease peri-operative cytokines levels in patients at high risk of post-operative complications.

## Methods

### Study design

This prospective single-centre randomized controlled trial took place in the Centre Hospitalier Universitaire Vaudois (CHUV), Lausanne, Switzerland, between May 2016 and January 2018. The target population included patients planned for elective cardiac surgery with expected long CPB duration and deemed at high risk of post-operative complications. The study protocol was approved by the Ethics Committee Vaud (2015-00010) and registered at ClinicalTrials.gov (NCT02775123).

#### Inclusion and exclusion criteria

To be eligible to enter the study, patients had to fulfil at least one of the following inclusion criteria: age > 75 years, double valve replacement, complex surgery with expected CPB duration > 120 min, redo cardiac surgery, pre-operative chronic renal failure (glomerular filtration rate < 30 ml/min) or chronic heart failure (< 40% left ventricular ejection fraction).

Exclusion criteria were end-stage renal disease (dialysis dependence), active infectious endocarditis, emergency or off-pump procedure, receipt of non-steroidal anti-inflammatory medication (except for low-dose aspirin) or corticosteroids within 7 days or enrolment in another conflicting study. Eligible patients were approached, and written informed consent was obtained prior to randomization.

#### Randomization

Randomization sequence was created using the Excel (Microsoft, Redmond, USA) Rand () function with a 1:1 ratio and permuted blocks of random sizes (1–5). Allocation information was stored within sealed opaque and numbered envelopes.

#### Blinding

Patients, surgical, anaesthetic and ICU teams as well as laboratory staff were all blinded regarding group allocation. Only the perfusionist managing the CPB and a co-investigator were aware of the patient’s allocation. All reasonable efforts were made to conceal the device (or absence of) from the sight of anaesthesiologists and surgeons; however, no sham device was used for control cases.

#### Primary outcome

Our primary outcome was the change in blood levels of key cytokines (IL-1α, IL-1β, IL-2, IL-4, IL-5, IL-6, IL-10, IFN-γ, MCP-1 and TNFα) in the peri-operative period according to whether or not CytoSorb® was inserted in the CPB. Such cytokines were measured in both groups after induction of anaesthesia (T0), at the end of CPB (after protamine administration; T2), 6 h after CPB initiation (T3) and 24 h after CPB initiation (T4).

#### Secondary outcomes

##### Safety

The impact of CytoSorb® HA on coagulation factors’ activity [antithrombin (AT), von Willebrand factor (vWF), factors II, V, VIII, IX, XI and XII] was assessed through measurements performed at the aforementioned time points in a subset of 21 consecutive patients (HA; *n* = 10, control; *n* = 11). For patients allocated to the HA group, an additional measure was performed at T1 (60 min after CPB initiation) to evaluate cross-adsorber coagulation factors’ clearance. For this measure, simultaneous samples were collected from the access (venous) line (pre-CPB) and the return (arterial) line (post-CPB). Baseline and perioperative characteristics as well as their outcomes of patients included in this sub-study are presented in Additional file 1: Tables S1 and S2.

Adverse events (AE) occurring within 28 days of randomization were systematically collected for each patient and assessed for potential association with the intervention. A serious adverse event was defined as any untoward medical occurrence that results in death, is life threatening, required prolonged hospitalization, results in persistent or significant disability or requires intervention to prevent impairment or damage.

##### Efficacy

In addition to our primary outcome measures, the following clinical outcomes were assessed: vasopressor and inotropic support (need for any vasoconstrictor or inotrope) within 24 h of ICU admission, need for any mechanical assistance (IABP, ECMO), mechanical ventilation duration, fluid balance, incidence of AKI (according to KDIGO criteria [16]), need for post-operative renal replacement therapy and ICU length of stay.

### Data collection

Baseline pre-operative characteristics (age, sex, body weight and chronic diseases), chronic medications (aspirin, angiotensin-converting enzyme inhibitors and angiotensin II receptors blockers), routine laboratory values and data relevant to the undertaken surgical procedure (type, CPB characteristics and fluid balance) were collected. To be considered, pre-operative values had to be obtained within 24 h of the procedure and discharge values had to be the latest obtained before discharge. Post-operative values for haemoglobin, platelets, aPTT and PT were obtained on ICU admission. Outcomes’ data were collected at ICU and hospital discharge.

### Procedure

General anaesthesia was induced with propofol, sufentanil and rocuronium. It was maintained with sufentanil boluses, sevoflurane before and after bypass and propofol during bypass. Tranexamic acid was administered as a 10-mg/kg bolus on anaesthesia induction followed by a 1-mg/kg/h infusion throughout CPB. Heparinization was initiated with a 300-UI/kg bolus and titrated according to haemostasis management system (HMS) Plus (Medtronic, Minneapolis, USA). An ACT > 400 s was required to initiate CPB. At the end of the procedure, heparin was reversed with protamine titrated according to HMS calculations.

The CPB included a Capiox® FX25 (Terumo, Tokyo, Japan) or Quadrox-i® (Maquet, Rastatt, Germany) membrane oxygenator and a heater-cooler system (Livanova, London, England). A roller or centrifugal pump was used according to the perfusionist’s preferences. The circuit was primed with heparin and Plasmalyte-A® (Baxter, Deerfield, USA). If deemed necessary, a haemofilter (haemoconcentrator, Sorin) was used. A non-pulsatile flow at 2.4 L/m^2^/min was maintained with a target mean arterial pressure between 60 and 80 mmHg. For this purpose, phenylephrine was administrated if deemed necessary. Myocardial protection was achieved using either St. Thomas® (Bichsel Laboratorium, Interlaken, Switzerland) or Custodiol® HTK (Sandor Medicaids, Hyderabad, India) cardioplegia solution, according to the surgeon’s preferences. Transesophageal echocardiography (TEE) was performed throughout the procedure.

For patients allocated to the HA group, a CytoSorb® cartridge was purged with NaCl 0.9% and integrated into the CPB circuit. The device was inserted in a side arm connected to the outflow line (high-pressure section) and the venous reservoir, prior to the oxygenator. No flow monitoring was conducted.

### Blood sampling

Blood samples were drawn in ethylene-diamine-tetra-acetic acid (EDTA) (cytokine analyses) or 0.106 M sodium citrate (coagulation factors) tubes. All samples were collected using standard hygiene precautions from patients’ arterial line or within the CPB circuit (T1 pre and post-adsorber measurements).

EDTA samples were centrifuged at 1500*g* at 4 °C for 10 min. Three aliquots of 100-μl plasma were prepared and stored at − 80 °C. Cytokine quantification was performed by the Mouse Metabolic Evaluation Facility (MEF), University of Lausanne, using Human Cytokine Magnetic 10-Plex Panel on the Luminex® Platform (Thermo Fischer Scientific®, Waltham, USA) [17]. Detection thresholds for cytokines are presented in Additional file 1: Table S3.

Citrate samples were centrifuged at 1500*g* for 15 min. Plasma samples were then preserved at − 20 °C. For analyses, samples were thawed at 37 °C in 10 min. Residual heparin activity was reversed with protamine according to measured anti-Xa activity (1 UI per UI/ml anti-XA activity). Factor II, V, VIII, IX, XI and XII activity was measured using a one-stage assay method. AT and vWF activity were assessed using a chromogenic method (respectively Berichrom® Antithrombin III, and INNOVANCE® vWFAc). Coagulation factors’ activity measurements were all performed at our central coagulation laboratory, using Sysmex® CS-5100 System (Siemens Healthineers, Erlangen, Germany).

### Statistical analysis

Based on previous literature [18], we calculated that, in order to be able to demonstrate a 25% change in serum IL-6 concentration, assuming a mean value of 200 pg/ml and a standard deviation of 50 pg/ml, we would need to recruit 15 patients in each arm to achieve a power of 80% with statistical significance set at 0.05.

A continuous variable is reported using mean and standard deviation (SD) or median and interquartile range (IQR) according to data distribution. Categorical variables are described using absolute frequencies and relative percentages.

For baseline characteristics and clinical outcomes comparisons, differences between groups were analysed by using *t* test or Wilcoxon signed-rank test for continuous variables and Fisher’s exact test for categorical variables. A *p* value < 0.05 was considered statistically significant.

For cytokine level comparisons, since cytokine levels were non-normally distributed, they are reported as median (IQR). The Mann-Whitney *U* test was performed for inter-group comparisons at each time point. Effect of time within each group was assessed using the Kruskal-Wallis one-way analysis of variance. A *p* value < 0.05 was considered statistically significant.

For coagulation factors’ analyses, comparisons of pre- and post-adsorber measurements of coagulation factors’ activity were performed using paired Student’s *t* tests. The effect of HA on coagulation factors throughout study time points was assessed by repeated measures’ analysis of variance (ANOVA) and analysis of covariance (ANCOVA) models. Final ANCOVA models included FFP administration, fluid balance and baseline value. Statistical threshold was determined as *p* = 0.016 after Bonferroni correction.

All analyses were performed using IBM SPSS Statistics (25).

## Results

During the study period, 569 patients underwent elective cardiac surgery with CPB in our institution. Of those, 211 (37%) were considered at high risk of post-operative complications and were potentially eligible to participate in this study. Altogether, 33 patients were enrolled in the trial. Of those, three were excluded: one withdrew his consent prior to the intervention, one patient had a last minute change of the surgical plan and one had an intra-operative unexpected discovery of an infective endocarditis. Hence, 15 patients were assigned to the control group and 15 to the HA group.

### Baseline demographics and peri-operative characteristics

Baseline and peri-operative characteristics of included patients are detailed in Table 1. There was no difference between the two groups in terms of baseline or peri-operative characteristics except for a lower protamine/heparin ratio in the HA group (*p* = 0.02). Median CPB duration was 142.5 (114.3–200) min.

**Table 1:** Baseline and peri-operative characteristics*

	Control (*N* = 15)	CytoSorb (*N* = 15)
Pre-operative characteristics		
Median age—(IQR) years	69 (49–80)	67 (44–76)
Male sex—no. (%)	11 (73.3)	13 (86.7)
Median body weight—(IQR) kg	80 (76–94)	86 (73–91)
Median left ventricular ejection fraction—(IQR) %	60 (35–65)	53 (43–57)
Median Euroscore II—(IQR) %	5.1 (2.1–7.2)	3.0 (2.2–9.1)
Coexisting conditions—no. (%)		
Chronic kidney disease^†^	4 (26.7)	3 (20)
Chronic heart failure ^‡^	5 (33.3)	2 (13.3)
Hypertension^±^	9 (60.0)	10 (66.7)
Diabetes^±^	2 (13.3)	4 (26.7)
Cerebro-vascular disease^∞^	1 (6.7)	0 (0)
Peripheral vascular disease	4 (26.7)	3 (42.9)
Smoking	6 (40)	7 (46.7)
Chronic obstructive pulmonary disease^£^	2 (13.3)	1 (6.7)
Mean pre-op haemoglobin level—(SD) g/L	134 (19)	132 (19)
Mean pre-op creatinine level—(SD) μmol/L	104 (34)	89 (22)
Intraoperative characteristics		
Type of procedure—no. (%)		
CABG or single valve replacement	2 (13.3)	2 (13.3)
Double valve replacement	1 (6.7)	0 (6.7)
CABG and valve replacement	6 (40)	3 (20)
Ascending aortic procedure	5 (33.3)	5 (33.3)
Others	1 (6.7)	5 (33.3)
Cardio-pulmonary bypass characteristics		
Median bypass duration—(IQR) min	138 (87–207)	145 (130–183)
Median cross-clamp duration (IQR) min	115 (68–159)	122 (97–146)
Centrifugal pump—no. (%)	5 (33.3)	4 (26.7)
Ultrafiltration—no. (%)	9 (45)	11 (73.3)
Median ultrafiltration volume—(IQR) mL	1000 (0–2500)	1500 (0–2000)
Modified ultrafiltration—no. (%)	2 (13.3)	2 (13.3)
Modified ultrafiltration volume—(IQR) mL	0 (0–0)	0 (0–0)
Fluid Balance at T2—(IQR) mL	2840 (2105–3643)	3125 (2480–3946)
Fluid Balance at T3—(IQR) mL	4371 (2921–5181)	4324 (3556–5460)
Fluid Balance at T4—(IQR) mL	6551 (5139–7301)	6702 (5657–7332)
Median SAPS II on ICU admission (IQR)	37.0 (34.0–49.0)	43.0 (21.0–48.0)

*CABG* coronary artery bypass grafting, *IQR* interquartile range, *SAPS II* simplified acute physiology score, *SD* standard deviation, *ICU* intensive care unit

*There were no significant differences between the groups with regard to any pre-operative or intra-operative characteristics (all *p* values > 0.05)

^†^Chronic kidney disease was defined by a creatinine clearance < 30 ml/min

^‡^Chronic heart failure was defined by a left ventricular ejection fraction < 40%

^±^Hypertension and diabetes were defined as the need for a disease specific medication

^∞^Cerebro-vascular disease was defined by a history of stroke or transient ischemic attack

^£^COPD was defined by a documented FEV1/FVC < 0.7 according to the GOLD criteria

### Primary outcome: cytokine levels

**Fig. 1:**
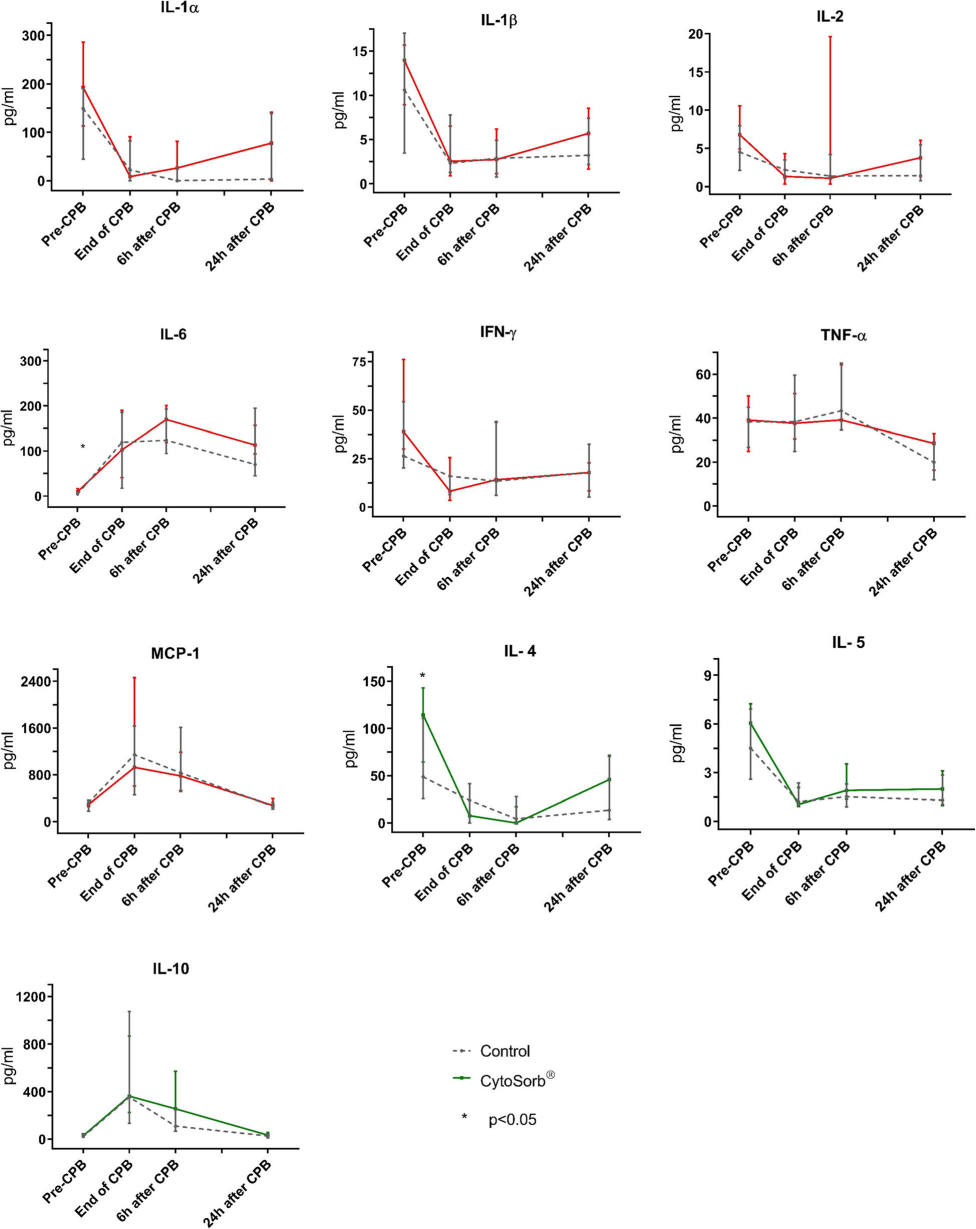
Median pro-inflammatory cytokine measurements throughout the study period. Whiskers indicate IQR. T0, induction of anaesthesia; T2, end of cardio-pulmonary bypass; T3, 6 h after the end of cardio-pulmonary bypass; and T4, 24 h after the end of CPB. IL interleukin; IFN-γ, interferon-gamma; MCP-1, monocyte chemoattractant protein-1; TNF-α, tumour necrosis factor-alpha. Inter-group comparisons performed using the Mann-Whitney *U* test comparisons at each time point. Effect of time within each group was significant (Kruskal-Wallis one-way analysis of variance) for all measurements (*p* < 0.05) except for IFN-γ in the control group (*p* = 0.09) and IL-2 in the control (*p* = 0.232)

Cytokines’ measurements throughout study time points are presented in Fig. 1. Il-6 and Il-4 baseline serum levels were higher in the HA group. Compared to baseline values, the surgical procedure was associated with an increase in IL-6 (peak level 6 h after CPB initiation, T3), MCP-1 and IL-10 levels (peak level at the end of CPB, T2). On the other hand, it was associated with a decrease in IL-1α, IL-1β, IL-2, IL-4, IL-5 and IFN-γ levels (nadir at T2 and T3). No change in TNF-α levels was observed until T4. There was no statistically significant difference between the two groups in serum levels of any of the cytokines of interest at any time point.

### Clinical outcomes

Clinical outcomes are reported in Table 2. There was no significant difference between the two groups in the use of vasoconstricting drugs (*p* = 1.0), mean noradrenaline dose on the first post-operative day (*p* = 0.87), use of any post-operative inotrope (*p* = 0.68), duration of mechanical ventilation (*p* = 0.31), incidence of AKI (*p* = 1.0), ICU length of stay (*p* = 1.0) and ICU and hospital mortality (both *p* = 1.0).

**Table 2:** Clinical outcomes*

	Control (*N* = 15)	CytoSorb (*N* = 15)
Outcome		
Re-operation within 48 h—no. (%)	1 (6.7)	0 (0)
Post-operative extracorporeal membrane oxygenation—no. (%)	0 (0)	1 (6.7)
Post-operative intra-aortic balloon pump—no. (%)	0 (0)	0 (0)
Vasoconstrictors		
Need for any vasoconstrictor—no. (%)	13 (86.7)	14 (93.3)
Median noradrenaline dose post-operative day 1—(IQR) μg/min	4.8 (2.0–13.7)	5.6 (1.5–6.8)
Median noradrenaline dose post-operative ICU stay—(IQR) μg/min	4.3 (2.0–7.8)	4.7 (1.5–7.1)
Inotropes		
Need for any inotrope—no. (%)	12 (80.0)	10 (66.7)
Median dobutamine dose post-operative day 1—(IQR) μg/min	0 (0)	0 (0–162)
Median dobutamine dose post-operative ICU Stay—(IQR) μg/min	0 (0–103)	0 (0–141)
Fluid balance at 24 h ICU—(IQR) ml	2240 (400–3589)	3000 (2000–4250)
Diuresis 24 h ICU—(IQR) ml	1125 (895–1700)	1495 (970–1930)
Mechanical ventilation—(IQR) hours	8 (2–102)	5 (0–16)
Acute kidney injury—no. (%)†	4 (26.7)	4 (26.7)
Post-operative need for renal replacement therapy—no. (%)	1 (6.7)	0 (0)
Renal replacement therapy dependence on hospital discharge—no. (%)	1 (6.7)	0 (0)
Serum creatinine on ICU discharge—(IQR) μmol/l	78 (65–126)	73 (68–103)
Serum creatinine on hospital discharge—(IQR) μmol/l	87 (72–120)	78 (68–97)
ICU length of stay—(IQR) days	1.0 (0.9–8.9)	1.8 (0.9–2.0)
Hospital length of stay—(IQR) days	12.0 (11.0–17.0)	12.5 (6.0–19.0)
ICU readmission—no. (%)	1 (7.7)	2 (13.3)
ICU mortality—no. (%)	2 (13.3)	1 (6.7)
Hospital mortality—no. (%)	2 (13.3)	1 (6.7)

*ICU* intensive care unit, *IQR* interquartile range, *SD* standard deviation

*There were no significant differences between the groups with regard to any pre-operative or intra-operative characteristics (all *p* values > 0.05)

^†^Acute kidney injury was defined as per the Kidney Disease: Improving Global Outcomes (KDIGO) classification

### Safety

#### Coagulation factors

##### Cross-adsorber clearance

**Fig. 2:**
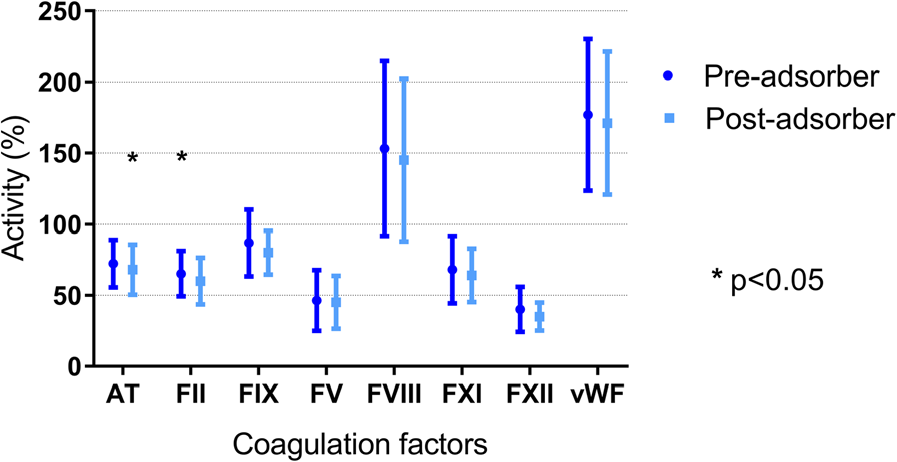
Coagulation factors cross-adsorber clearance. Pre- and post-adsorber samples were collected 60 min after the initiation of CPB (T1). AT, antithrombin; F, factor; vWF, von Willebrand factor. Comparisons performed using paired Student’s *t* tests

Pre- and post-adsorber measurements of coagulation factors’ levels are reported in Fig. 2. There was no statistically significant difference in factors V, VIII, IX, XI and XII nor in vWF activity between pre- and post-adsorber measurements. A small but statistically significant decrease in AT and FII was observed (resp. from 70.4 ± 15.3 to 66.6 ± 16.5, *p* = 0.006, and 61.6 ± 16.2 to 57.3 ± 16.3, *p* = 0.03).

##### Time comparisons

**Fig. 3:**
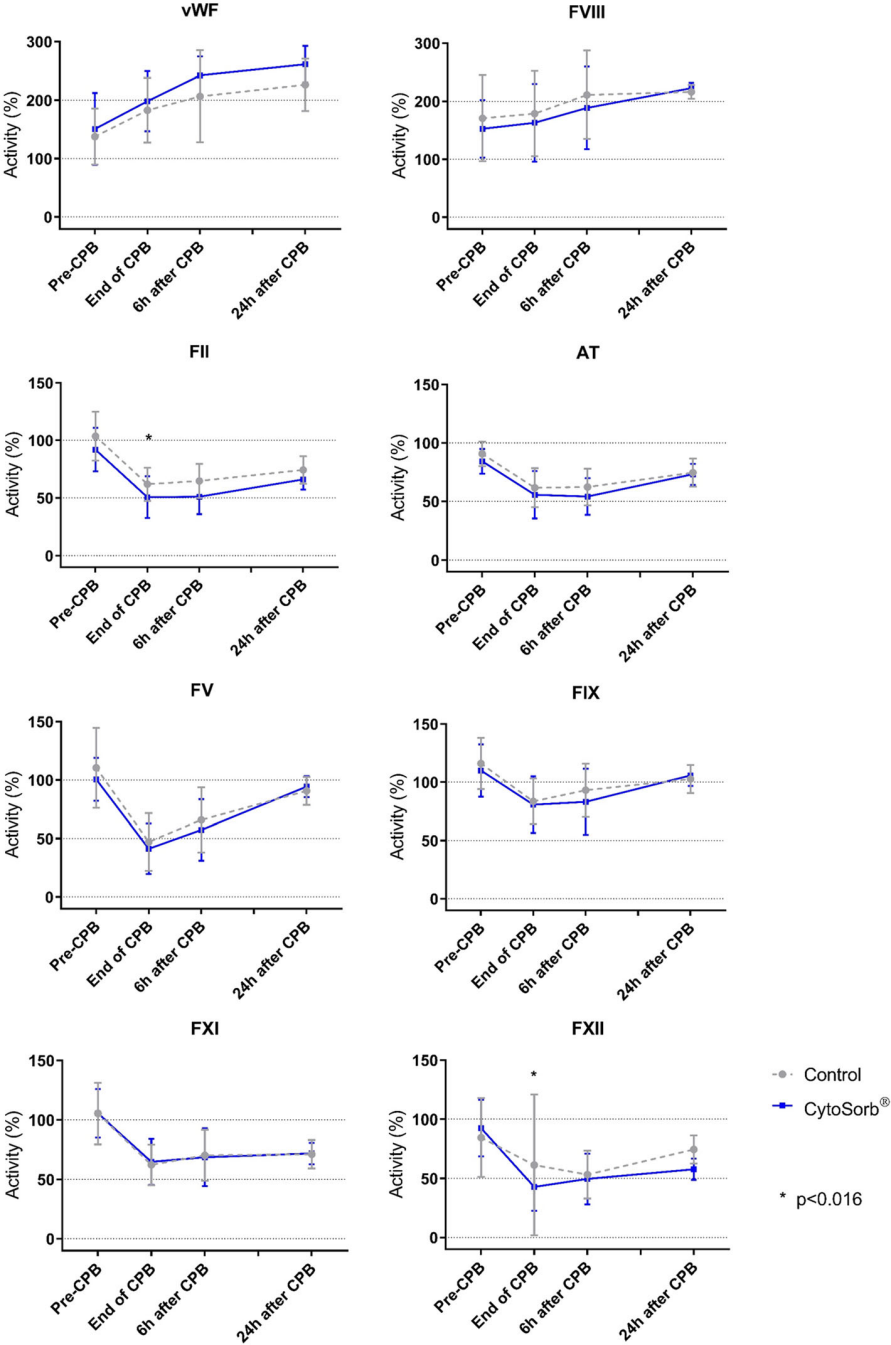
Coagulation factors measurements throughout the study period. T0, induction of anaesthesia; T2, end of CPB; T3, 6 h after the end of CPB; and T4, 24 h after the end of CPB. AT, antithrombin; F, factor; vWF, von Willebrand factor. The effect of HA on coagulation factors throughout study time points was assessed by repeated measures’ analysis of variance (ANOVA) and analysis of covariance (ANCOVA) models. Final ANCOVA models included FFP administration, fluid balance and baseline value. Statistical threshold was determined as *p* = 0.016 after Bonferroni correction

The evolution of coagulation factors throughout the study period is presented in Fig. 3. Unadjusted analyses revealed a difference in vWF and factor II activities (*p* = 0.04 and 0.005); however, after adjustment for baseline values, FFP and fluid balance, only factor II activity remained statistically significantly lower after the end of CPB in the HA group (*p* = 0.02). In addition, adjusted analyses revealed a lower factor XII activity at the end of CPB in the HA group (*p* = 0.005).

##### Classic haematological parameters

Pre- and post-operative values for haemoglobin, thrombocytes, aPTT and INR are presented in Additional file 1: Table S4. There was no statistically significant difference between the two groups for these parameters.

#### Other adverse events

As summarized in Table 3 (and detailed in Additional file 1: Table S5), 53 AE were reported during the study: 30 (10 patients) in the control group and 23 (11 patients) in the CytoSorb group (*p* = 1.0 for number of patients). Of those, 25 were considered as serious AE: 12 (eight patients) in the control group and 13 (eight patients) in the CytoSorb group (*p* = 1.0 for number of patients). Fatal AE were reported for two (13.3%) patients in the control group and one (6.7%) in the CytoSorb group (*p* = 1.0). Cause of death is detailed in Additional file 1: Table S6. Categories of encountered AE and respective distribution between the two groups are presented in Table 3. No obvious AE appeared to be directly attributable to the CytoSorb® device.

**Table 3:** Adverse events and serious adverse events

	Control (*n* = 15)	CytoSorb (*n* = 15)	*p* value
Total adverse events	30	23	
Fatal adverse events	2	1	
Patients with fatal adverse event—no. (%)	2 (13.3)	1 (6.7)	1.00
Patients with severe non-fatal adverse event—no. (%)	8 (53.3)	8 (53.3)	1.00
Patients with at least 1 adverse event—no. (%)	10 (66.7)	11 (73.3)	1.00
Adverse events categories			
Respiratory—no. (%)	0 (0)	2 (13.3)	0.48
Cardiogenic shock—no. (%)	0 (0)	1 (6.7)	1.00
Haemorrhagic shock—no. (%)	0 (0)	1 (6.7)	1.00
Distributive shock—no. (%)	2 (13.3)	1 (6.7)	1.00
Arrhythmias—no. (%)	8 (53.3)	5 (33.3)	0.46
Surgical complications—no. (%)	4 (26.7)	4 (26.7)	1.00
Infection—no. (%)	4 (26.7)	1 (6.7)	0.33
Acute liver failure—no. (%)	0 (0)	1 (6.7)	1.00
Acute kidney injury—no. (%)	4 (26.7)	4 (26.7)	1.00
Neurological AE (including stroke)—no. (%)	3 (20.0)	2 (13.3)	1.00
Electrolyte disorders—no. (%)	1 (6.7)	0 (0)	1.00

All adverse events documented in the first 28 days following the randomisation were recorded. All patients who died were analysed and classified as fatal serious events. Patients were counted once for each event category even if they had multiple events in that category. Comparisons were made by Fisher’s exact test

## Discussion

### Key findings

We performed a single-centre pilot randomized controlled trial on 30 patients undergoing elective cardiac surgery and deemed at high risk of peri-operative complications. In those patients, we evaluated the safety and efficacy of CytoSorb® haemoadsorption during CPB in comparison with standard management. We found that the procedure, as described, appeared safe and feasible as no immediate or delayed complication attributable to the device was observed. A thorough investigation of coagulation parameters did not demonstrate any relevant alteration of coagulation factors or thrombocytes levels associated with the procedure. However, the intervention was not associated with a decrease in key cytokine levels. Similarly, although the study was not powered to examine these outcomes, it was not associated with a difference in terms of need for vasoconstrictors, post-operative AKI or need for RRT, ICU length of stay and in-hospital mortality.

### Comparison with previous studies

Our main result is consistent with findings from a previous and similar RCT conducted in Austria by Bernardi et al. [15]. In this trial, patients undergoing elective cardiac surgery with an expected CPB duration of more than 120 min were randomly allocated to either CytoSorb® HA or control group. After the exclusion of five patients, 32 were included in the main analysis (16 in each group). The authors did not find any difference in peri-operative levels of IL-6, IL-10, IL-18, IL-1β and TNF-α and HMGβ1 except for a longer decay for IL-10 in the CytoSorb® group.

Patients included in both trials appear to be comparable in terms of age, EuroSCORE and comorbidities. Although arguably patients included in our study underwent more complex procedures (aortic procedures), this did not translate in a longer CPB duration (138/145 min versus 170/191 min). Hence, both studies have evaluated CytoSorb® HA in a collective of patients at high risk of complications.

We have analysed a different panel of cytokines, including pro-inflammatory IL-1α, IL-1β, IL-6, IFNγ, TNFα and MCP-1, as well as anti-inflammatory, IL-4, IL-5 and IL-10. However, similar to Bernardi et al., we failed to demonstrate any effect of CytoSorb® HA on these cytokines. Of note, we did not observe higher IL-10 levels in the CytoSorb group. The absence of efficacy observed in the Austrian trial [15] is therefore confirmed by our data. In addition, consistent with the observation made, we have observed large inter-patient variability.

The observed lack of efficacy of the therapy might be explained by the low inflammatory response observed in patients included in both trials. Indeed, in an observational study, intra-operative CytoSorb HA was associated with a decreased requirement for post-operative vasoconstrictors in 39 patients with infective endocarditis compared with historical controls [13]. However, in these patients, peak (post-operative) IL-6 levels were around 400 mg/ml, a value much higher than those observed in our trial (median peak IL-6 levels (2 h post-CPB) 120.8 [49.0–160.8] pg/ml in the HA group and 118.7 [68.4–255.9] pg/ml in the control group).

IL-6, IL-10 and MCP-1 have exhibited the classically reported pattern of an increase associated with the CPB followed by slow return to baseline. However, less commonly reported cytokines (IL-1 α, IL-1β, IL-4, IL-5) have exhibited a different pattern. In both groups, CPB was associated with a *decrease* in the plasma level of these cytokines. This pattern is an unexpected finding of unknown significance. It could be partly related to haemodilution but at a minimum suggests against a significant activation of their signalling systems induced by CPB.

To the best of our knowledge, this is the first trial evaluating the consequences of CytoSorb® HA on coagulation parameters. Several studies have reported a trend for thrombocytopenia [19] during HA. We have not observed such association.

### Strengths and limitations

This study has several strengths. Despite the small sample size, baseline characteristics were well balanced between the two groups of patients. We have examined a large panel of pro- and anti-inflammatory cytokines at different relevant time points. In addition, we have conducted a thorough evaluation of CytoSorb® effect on several coagulation parameters. These analyses involved cross-adsorber clearance measurement, coagulation factors’ levels throughout relevant time points and conventional haematological evaluation. To the best of our knowledge, our study is the first to provide such a broad assessment during CytoSorb® HA.

Some limitations are however present. First, it is a monocentric pilot study, on a rather small collective of patients. Our results are therefore subject to selection bias, and the external validity of our results can be limited. However, our findings are largely consistent with those obtained in a similar study but conducted in another health care system and another country.

Second, the duration of CytoSorb® HA was restricted to the CPB duration, which might not be sufficient to demonstrate efficacy. Indeed, cytokine release requires complex intracellular signalling mechanisms before they can be upregulated, expressed and secreted. The device insertion might have been too early relative to CPB-induced immune response and the duration of the therapy insufficient. However, insertion of the cartridge during CPB is a pragmatic, easy and potentially generalizable practice with minimal manipulations and risks to the patient. Longer therapy would require insertion of a double-lumen catheter and prolonged anticoagulation, which might not be desirable.

Third, although we have selected patients at risk of post-operative complications, the actual rate of such complications was relatively low and most patients required little ICU support. This might be consistent with the relatively low cytokine levels. A positive effect of the device on patients with higher inflammatory response and higher incidence of post-pump syndrome cannot be ruled out.

Finally, the utilization of modified ultrafiltration in some patients might have attenuated the effect of the device [20]. However, such utilization was limited to two patients in each group. Post hoc analyses performed after exclusion of these patients confirmed main results.

### Study implications

Our study shows that, even in patients at high risk of complications, as defined by clinical criteria, CytoSorb® HA, during CPB is not associated with a significant decrease in cytokine levels. This absence of effect might be related to a low inflammatory response, to an insufficient therapy duration or to an inadequate timing relative to pro-inflammatory cytokine production. Although an effect in patients with higher rate of post-pump syndrome and higher inflammatory response cannot be ruled out, CytoSorb® HA is not likely to be beneficial in the vast majority of elective cardiac procedures with low to moderate inflammatory responses. Together with the trial from Bernardi et al. [15], and despite small sample sizes, we can now conclude that routine application of CytoSorb® HA seems not to be justified for elective cardiac procedures. Indeed, given the absence of effect in two well-conducted RCT in patients with high risk of complications and prolonged CPB but nevertheless low cytokine levels, it is highly unlikely that a larger or multi-centre trial would demonstrate a benefit.

Further studies are required to evaluate its potential in particular situations perhaps associated with higher cytokine levels such as emergency procedures, acute infectious endocarditis or heart transplantation as suggested by observational studies [12, 13]. Post-operative use of CytoSorb might also need to be evaluated [20]. The observed heterogeneity of peri-operative measurements might suggest that some particular patients could benefit from the therapy. Hence, further studies might attempt to include a point-of-care evaluation of cytokine levels to enrich study population and restrict the intervention to patients with elevated cytokine levels.

On the other hand, our trial has provided important safety data in particular regarding the coagulation system. The absence of significant alteration of the coagulation system during the therapy is of major interest. The small but significant decrease in AT and FII across the adsorber might be interpreted as coagulation activation. Together with the decreased protamine to heparin ratio, reflecting known heparin adsorption in the HA device and the decrease FXII activity at the end of CPB reflecting contact activation, our findings further validate the need for therapeutic anticoagulation associated with the procedure.

## Conclusions

In patients at risk of post-operative complications, CytoSorb® haemoadsorption during cardiac surgery was not associated with a decrease in cytokine levels or an improvement in relevant clinical outcomes. However, the procedure appeared safe and feasible. In particular, a thorough evaluation of coagulation profiles did not reveal any significant alterations in conventional haematological parameters or coagulation factors’ levels. Further studies are required to evaluate CytoSorb® HA utilization in situations associated with very high inflammatory response or in established post-pump syndrome.

## Additional file

Additional file 1: **Table S1.** Baseline and peri-operative characteristics of patients included in the coagulation sub study. *There were no significant differences between the groups with regard to any pre-operative or intra-operative characteristics (all *p* values > 0.05) except for protamine/heparin ratio (*p* = 0.02). †Chronic kidney disease was defined by a creatinine clearance < 30 ml/min. ‡Chronic heart failure was defined by a left ventricular ejection fraction < 40%. CABG, coronary artery bypass grafting, IQR interquartile range, SAPS II simplified acute physiology score, SD standard deviation, ICU intensive care unit. **Table S2.** Outcomes of patients included in the coagulation sub study. **Table S3.** Minimal detection range of cytokines by the Luminex® Platform. **Table S4.** Haemoglobin, platelets and coagulation tests. To be considered, pre-operative values, had to be obtained within 24 h of the procedure. Post-operative values were obtained on ICU admission. Abbreviations: aPTT activated partial thromboplastin time, INR international normalized ratio. *p* value for the effect of group in ANOVA for repeated measures. **Table S5.** Detailed list of adverse events. Table S6. Cause of death. (RTF 370 kb)

## Abbreviations

AE: Adverse events
AKI: Acute kidney injury
ANCOVA: Analysis of covariance
ANOVA: Repeated measures analysis of variance
aPTT: Partial thromboplastin time
AT: Antithrombin
CPB: Cardio pulmonary bypass
EDTA: Ethylene-diamine-tetra-acetic acid
FFP: Fresh frozen plasma
HA: Haemoadsorption
HMS: Haemostasis management system
ICU: Intensive care unit
IQR: Interquartile range
KDIGO: Kidney Disease: Improving Global Outcomes
PT: Prothrombin time
vWf: von Willebrand factor
