# Supplementary material for: Cytokine clearance with CytoSorb® during cardiac surgery: a pilot randomized controlled trial
Source: Crit Care. 2019 Apr 3;23:108. doi: 10.1186/s13054-019-2399-4 (PMC6448322; doi:10.1186/s13054-019-2399-4)
Supplement: Supplementary file 1 — Table S1. Baseline and peri-operative characteristics of patients included in the coagulation sub study. *There were no significant differences between the groups with regard to any pre-operative or intra-operative characteristics (all p values > 0.05) except for protamine/heparin ratio (p = 0.02). †Chronic kidney disease was defined by a creatinine clearance < 30 ml/min. ‡Chronic heart failure was defined by a left ventricular ejection fraction < 40%. CABG, coronary artery bypass grafting, IQR interquartile range, SAPS II simplified acute physiology score, SD standard deviation, ICU intensive care unit. Table S2. Outcomes of patients included in the coagulation sub study. Table S3. Minimal detection range of cytokines by the Luminex® Platform. Table S4. Haemoglobin, platelets and coagulation tests. To be considered, pre-operative values, had to be obtained within 24 h of the procedure. Post-operative values were obtained on ICU admission. Abbreviations: aPTT activated partial thromboplastin time, INR international normalized ratio. p value for the effect of group in ANOVA for repeated measures. Table S5. Detailed list of adverse events. Table S6. Cause of death. (RTF 370 kb) [file 13054_2019_2399_MOESM1_ESM.rtf]

	Control
N=11	CytoSorb
(N=10)	
Peri-operative characteristics			
 Median Age – (IQR) years	69 (49-80)	67 (44-76)	
Median body weight – (IQR) kg	8 (73%)	8 (80%)	
Body Weight ç	81 (78-98)	76 (71-91)	
Median left ventricular ejection fraction – (IQR) %	60 (35-65)	54 (42-57)	
Median Euroscore II – (IQR) %	2.5 (1.6-10.2)	3.0 (2.3-7.2)	
Co-existing conditions – no. (%)			
Chronic kidney disease†	4 (36%)	2 (20%)	
Chronic heart failure ‡	5 (45%)	2 (20%)	
Diabetes	2 (18%)	4 (40%)	
Hypertension	7 (64%)	6 (60%)	
Cerebro-vascular disease	1 (9%)	0 (0%)	
Peripheral vascular disease	4 (36%)	1 (10%)	
Mean pre-op creatinine level – (SD) mmol/L	110 (38)	88.2 (24.7)	
Mean pre-op hemoglobin level – (SD) g/L	136 (21)	135.5 (19.6)	
Mean Pre op Trombocyte level (-(SD) G/L	221 (75)	217.6 (78)	
Mean pre-op INR – (SD)	1.0 (0)	1.02 (0.04)	
Mean pre-op aPTT – (SD)	35 (9.0)	31.2 (8.4)	
Intra-operative characteristics 			
Type of procedure – no. (%)			
CABG or single valve replacement	2 (18.1)	2 (18.2)	
Double valve replacement	1 (9.1)	0 (0)	
CABG and valve replacement	5 (45.4)	2 (20.0)	
Ascending aortic procedure	3 (27.3)	4 (40.0)	
Others	0 (0)	2 (20.0)	
Cardio-pulmonary bypass characteristics			
Median Bypass Duration - (IQR) min	135 (72-201)	143 (130-183)	
Median cross-clamp duration (IQR) min	107 (57-159)	110 (85-145)	
Ultrafiltration – no. (%)	7 (63%)	8 (80%)	
Ultrafitration Volume ç	1000 (0-2000)	1250 (480-2000)	
Modified UF	2 (18%)	2 (20%)	
Median ultrafiltration volume – (IQR) mL	0 (0-50)	0 (0-0)	
Centrifugal pump– no. (%)	4 (36%)	4 (40%)	
Median total heparin administered - (IQR) x1000 UI 	40 (32.7-50)	43.75 (35-55)	
Median total protamin administered - (IQR) x1000 UI	26 (22-32)	18.5 (11-26.1)	
Protamin/heparin ratio	0.75 (0.52-0.80)	0.42 (0.33-0.55)	
Fluid Balance at T4 - (IQR) mL	6792 (5523-9013)	6670 (5657-7041)	
Median blood transfusions (T0 to T4 – (IQR) no	0 (0-5)	1 (0-2)	
Median thrombocytes tranfusions  (T0 to T4) – (IQR) no	0 (0-0)	0 (0-0)	
Median Fibrinogen tranfusions  (T0 to T4) – (IQR) no	0 (0-1)	0 (0-2)	

Table S1: baseline et peri-operative characteristics of patients included in the coagulation sub study
* There were no significant differences between the groups with regard to any pre-operative or intra-operative characteristics (all p values > 0.05) except for protamine/heparin ratio (p=0.02)
† Chronic kidney disease was defined by a creatinine clearance < 30 ml/min 
‡Chronic heart failure was defined by a left ventricular ejection fraction < 40%
CABG: coronary artery bypass grafting, IQR: interquartile range, SAPS II: simplified acute physiology score, SD: standard deviation, ICU: intensive care unit


	Control 
(n=11)	Cytosorb
(n=10)	P value	
Re-operation within 48hrs – no (%)	1 (9.1%)	0 (0%)	1.00	
Post op ECMO – no(%)	0 (0%)	1 (10%)	0.45	
Acute kidney injury – no. (%)†	4 (36.4%)	1 (10%)	0.162	
Need for renal replacement therapy	1 (9.1%)	0 (0%)	1.00	
Need for any vasoconstrictor – no. (%)	10 (90.9%)	9 (90%)	1.00	
Need for any inotrope – no. (%)	8 (72.7%)	6 (60%)	1.00	
Death in ICU	2 (18.2%)	1 (10%)	1.00	
Death in Hospital	2 (18.2%)	1 (10%)	1.00	


Table S2: Outcomes of patients included in the coagulation sub study


Cytokine	Detection threshold 	
IFN-ã	0.8 pg/ml	
IL-10	8.6 pg/ml	
IL-1â	0.8 pg/ml	
IL-6	0.9 pg/ml	
MCP-1	1.9 pg/ml	
TNF-á	0.7 pg/ml	
IL-1á	9.4 pg/ml	
IL-2	1.0 pg/ml	
IL-4	4.5 pg/ml	
IL-5	0.5 pg/ml	


Table S3: Minimal detection range of cytokines by the Luminex ® Platform.


	Control (n=11)	CytoSorb (n=10)	P value	
	Baseline	ICU admission	Baseline	ICU admission		
Mean hemoglobin level – (SD) g/L	135.8 (21.2)	117.3 (19.9)	135.5 (19.6)	119.4 (21.9)	0.88	
Thrombocytes – (SD) giga/L	220.9 (74.8)	135.8 (49.9)	217.6 (78.5)	108.6 (51.2)	0.45	
aPTT - (SD) sec	34.8 (9.5)	45.3 (22.0)	31.2 (8.4)	46.1 (37.3)	0.84	
INR - (SD)	1.03 (0.05)	1.14 (0.07)	1.02 (0.04)	1.21 (0.24)	0.46	

Table S4: Hemoglobin, platelets and coagulation tests
To be considered, pre-operative values, had to be obtained within 24 hours of the procedure
Post- operative values were obtained on ICU admission
Abbreviations: aPTT: activated partial thromboplastin time, INR: international normalized ratio
P value for effect of group in ANOVA for repeated measures


Pt Nb	Group	Description	Category	Severe AE?	Lead to death?	
1	Control	Atrial flutter	Arrhythmia	0	0	
1	Control	Hospital acquired pneumonia	Infection	0	0	
1	Control	Vasoplegic shock	Vasoplegic shock	1	0	
1	Control	Delayed arousal	Neurological	0	0	
1	Control	Stage 2 AKI	AKI	0	0	
2	Control	Haemorrhagic shock	Surgical 	1	0	
2	Control	Stage 3 AKI	AKI	1	0	
2	Control	Ventricular tachycardia	Arrhythmia 	0	0	
2	Control	Atrial fibrillation	Arrhythmia	0	0	
2	Control	Ischemic stroke	Neurological	1	1	
2	Control	Sternum infection	Infection	0	0	
3	CytoSorb	3rd degree III AV Block	Arrhythmia	1	0	
5	Control	Atrial fibrillation	Arrhythmia	0	0	
5	Control	Hospital acquired pneumonia	Infection	0	0	
5	Control	Delirium	Neurological	0	0	
6	Control	Vasoplegic shock	Vasoplegic shock	1	0	
6	Control	Atrial fibrillation	Arrhythmia	0	0	
6	Control	3rd degree III AV Block	Arrhythmia	1	0	
6	Control	Ventilator acquired pneumonia	Infection	1	0	
6	Control	Stage 2 AKI	AKI	0	0	
8	CytoSorb	Acute liver failure	Acute liver failure	1	0	
8	CytoSorb	Stage 3 AKI	AKI	1	0	
8	CytoSorb	Haemorrhagic shock	Haemorrhagic shock	1	0	
8	CytoSorb	ARDS	Respiratory	1	0	
8	CytoSorb	Cardiogenic shock	Cardiogenic shock	1	1	
10	CytoSorb	Pleural lesion	Surgical	0	0	
13	CytoSorb	3rd degree III AV Block	Arrhythmia	1	0	
15	CytoSorb	Atrial fibrillation	Arrhythmia	0	0	
15	CytoSorb	Hospital acquired Pneumonia	Infection	0	0	
16	Control	Atrial fibrillation	Arrhythmia	0	0	
16	Control	Stage 1 AKI	AKI	0	0	
16	Control	Ventilator acquired Pneumonia	Infection	1	0	
16	Control	Severe hyponatremia 	Electrolytes	0	0	
17	Control	Atrial bradycardia 	Arrhythmia	1	0	
22	Control	Pericarditis	Surgical	0	0	
23	CytoSorb	3rd degree III AV Block	Arrhythmia	1	0	
23	CytoSorb	Atrial fibrillation	Arrhythmia	0	0	
26	CytoSorb	Atelectasis	Respiratory	0	0	
25	Control	Supraventricular tachycardia 	Arrhythmia	0	0	
25	Control	Pericardial effusion	Surgical	1	0	
26	CytoSorb	Stage 2 AKI	AKI	1	0	
26	CytoSorb	Vasoplegic shock	Vasoplegic shock	1	0	
27	CytoSorb	Right coronary lesion	Surgical	1	0	
28	Control	Right atrial lesion	Surgical	1	0	
30	CytoSorb	Pericardial effusion	Surgical	0	0	
30	CytoSorb	Stage 1 AKI	AKI	0	0	
31	CytoSorb	Stroke 	Neurological	1	0	
32	CytoSorb	Stage 1 AKI	AKI	0	0	
32	CytoSorb	Atrial fibrillation	Arrhythmia	0	0	
32	CytoSorb	Delirium	Neurological	0	0	
32	CytoSorb	Pericardial tamponade	Surgical	1	0	
33	Control	Ventricular tachycardia	Arrhythmia	1	0	
33	Control	Supraventricular Tachycardia 	Arrhythmia	0	0	

Table S5: Detailed list of adverse events


Patient Number	Group	Cause of death	
1	Control	Post-operative discovery of an undifferentiated multi-metastatic carcinoma. Palliative care decided. 	
2	Control	Massive ischemic stroke (two hemispheres and right cerebellum) discovered on post-op day 10.	
8	CytoSorb	Refractory hemorrhagic and distributive shock	

Table S6: Cause of death
